# A nomogram for predicting hospital-acquired venous thromboembolism in ICU patients with mechanical ventilation: a retrospective cohort study

**DOI:** 10.3389/fmed.2025.1653481

**Published:** 2025-11-17

**Authors:** Wenjie Ge, Aiqin Chu, Zhimin Cao, Xinyi Zhu, Shoujun Zhu

**Affiliations:** Department of Nursing, The First Affiliated Hospital of USTC, Division of Life Sciences and Medicine, University of Science and Technology of China, Hefei, Anhui, China

**Keywords:** critically ill, risk assessment model, mechanical ventilation, intensive care unit, hospital-acquired venous thromboembolism

## Abstract

**Objective:**

To develop a predictive nomogram for early identification of hospital-acquired venous thromboembolism (HA-VTE) in adult ICU patients undergoing mechanical ventilation.

**Methods:**

This study involved 472 ICU patients with mechanical ventilation in the Department of Intensive Care Unit of The First Affiliated Hospital of USTC from January 2021 to December 2022. The diagnosis of VTE was objectively confirmed by imaging studies. Clinical information and relevant laboratory test data were retrospectively collected. Logistic regression was utilized to pinpoint these patients’ independent risk factors for HA-VTE. Subsequently, a nomogram was established to predict HA-VTE risk. The efficacy of this model was assessed through the area under the receiver operating characteristic curve (AUC-ROC), alongside a calibration curve and the Hosmer–Lemeshow test to examine its fit. Additionally, decision curve analysis (DCA) was conducted to ascertain the clinical relevance of the predictive model.

**Results:**

The study incorporated 472 ICU patients with mechanical ventilation, with a HA-VTE rate of 12.50% (59 cases). Six independent predictors were identified and integrated into a predictive nomogram: stroke, bedridden for at least 3 days, caprini risk score, Glasgow Coma Scale, fibrinogen, and d-dimer. The nomogram demonstrated intense discrimination (AUC 0.909, 95% CI: 0.859–0.958). The calibration curve closely aligned with the ideal curve, and the Hosmer–Lemeshow goodness-of-fit test yielded a χ^2^ value of 6.398 with a *p*-value of 0.603, verifying the model’s high calibration accuracy. Additionally, the DCA indicated that the model provides a net benefit across a wide range of decision thresholds from 0 to 0.99, underscoring its clinical utility. Internal validation yielded a concordance index of 0.909, indicating robust reliability.

**Conclusion:**

This study established a validated nomogram incorporating six readily accessible clinical predictors to stratify HA-VTE risk in mechanically ventilated ICU patients. The tool facilitates early intervention and personalized prophylaxis strategies.

**Implications for clinical practice:**

The nomogram provides doctors with a pragmatic, evidence-based instrument to enhance the prevention of hospital-acquired venous thromboembolism in critically ill individuals on mechanical ventilation. Facilitating focused surveillance and customized anticoagulation strategies can diminish HA-VTE-related morbidity and healthcare expenditures while enhancing patient outcomes.

## 1 Introduction

Hospital-acquired venous thromboembolism (HA-VTE), which includes hospital-acquired deep vein thrombosis (HA-VDVT) and hospital-acquired pulmonary embolism (HA-PE), is defined as a new venous thromboembolism (VTE) event that occurs during a hospital stay or within 90 days of discharge (1), is a preventable cause of hospital death (2), affecting millions globally per annum as a leading contributor to global disease burden. In developed countries, it affects 1–2 per 1,000 persons, making it the third most frequent cardiovascular disorder, following myocardial infarction and stroke (3). Studies have shown that VTE and pulmonary embolism incidence rates in Western developed countries are about 0.87–1.82/1,000 person-years and 0.45–0.95/1,000 person-years, respectively (4, 5). The incidence rate of HA-VTE is (6–87)/100,000 (6), and HA-VTE was associated with a nearly three-fold increased odds of death during hospitalization in a diverse patient cohort from 5 hospital systems (7). A large-scale retrospective study conducted at a major Chinese hospital revealed an overall HA-VTE prevalence of 0.296% among adult hospitalizations, accounting for nearly one-quarter (23.7%) of all diagnosed VTE events, the crude incidence rate demonstrated a marked upward trajectory, escalating from 0.75 per 1,000 patients in 2016 to 5.89 per 1,000 patients in 2022, HA-VTE was associated with prolonged hospitalization and increased mortality notably, the morbidity and mortality rate is 2.1% (8, 9). A recent review by Grosse et al suggested that VTE costs the U.S. health care system around $7 to $10 billion per year for around 350,000–475,000 medically treated incident cases, underscoring its economic burden (10). Furthermore, HA-VTE is a common complication for patients undergoing emergency internal medicine or surgical operations. It not only prolongs patients’ hospital stay and affects their quality of life, but also poses a threat to their safety (11). These findings underscore the growing clinical significance of HA-VTE globally and highlight the critical need for enhanced surveillance strategies and predictive tools for early identification of high-risk hospitalized patients.

Critically ill patients face a heightened risk of VTE due to a combination of general risk factors and specific risk factors related to the intensive care unit (ICU), such as sedation, immobilization, vasopressor use, or central venous catheters. A recent study suggested that 30.9% ICU patients experienced inpatient VTE (12). Mechanical ventilation is a relatively typical life support technique in the ICU. Approximately 50.0–70.0% of ICU patients require mechanical ventilation treatment to achieve therapeutic effects such as maintaining airway patency, oxygen supply, and airway aspiration, and to ensure the oxygen supply needed by vital organs (13). In a multicenter retrospective review from the VPS registry, mechanical ventilation and duration were independent risk factors for hospital-acquired VTE among critically ill patients (14). Multiple VTE risk assessment tools, such as the Caprini score (15), Padua score (16), and Rogers score (17), are widely used in clinical practice and effectively identify high-risk patients. However, each model was developed for distinct patient populations. Mechanically ventilated ICU patients are a particular subgroup with unique characteristics, such as PEEP and treatment regimens (18). The application of these models to predict the VTE risk of mechanically ventilated ICU Patients has significant limitations, because they can’t include all VTE risk factors in mechanically ventilated ICU patients. Therefore, risk assessment of mechanically ventilated patients for more aggressive prophylactic strategies should be considered to reduce the potential morbidity associated with untreated HA-VTE in this high-risk population.

Clinical prediction models are evaluation tools that focus on risk identification and management in the early stages of disease development (19). Although studies have been conducted to explore the factors associated with the occurrence of VTE in mechanically ventilated patients in the ICU, most of the studies have remained at the level of single factors and simple statistical analyses, and have lacked an integrated and comprehensive predictive model (18, 20). Recently, the nomogram has gained prominence as a precise, easy-to-use predictive instrument extensively employed in clinical settings. It renders multivariate regression analysis into a visual format, which helps quantify and assess the risk factors and likelihood of clinical event occurrences through cumulative scoring. This visual representation of statistical predictive models is instrumental in enabling clinicians to identify patients at high risk quickly and supports the creation of targeted interventions (21). Therefore, this study is focused on creating a nomogram model to evaluate the HA-VTE risk in mechanically ventilated ICU patients. Utilizing this model allows for a more precise assessment of all-cause VTE risk within this demographic, facilitating the development of early intervention strategies customized to their specific needs, thereby enhancing life quality and reducing HA-VTE rates effectively.

## 2 Materials and methods

### 2.1 Study design and patients

This was a single-center, retrospective, cohort study. Adult patients who were admitted to the Intensive Care Unit (ICU) of the First Hospital of the University of Science and Technology of China and received mechanical ventilation support between January 2021 and December 2022 were included in the study. Those patients who were mechanically ventilated for < 24 h were excluded from the study. The criteria for inclusion involved: (1) age ≥ 18 years; (2) VTE diagnosed by vascular ultrasound, magnetic resonance imaging, or angiography during hospitalization; (3) Venous thrombosis from 48 h after mechanical ventilation to 48 h after extubation. The exclusive criteria were as follows: (1) Discharged for interruption of treatment for own reasons; (2) died within 48 h after admission; (3) Other systemic diseases that cause abnormal blood clotting mechanisms, such as cirrhosis, cardiolipin syndrome, hemophilia and other diseases and those taking anticoagulant drugs; and (4) incomplete clinical data. Ultimately, 472 patients were included in our study.

Ethical approval for this study was obtained from the Ethics Committee of The First Affiliated Hospital of USTC (Approval Number: 2023KY Ethics Review No.123). Additionally, the study adhered to the standards outlined in the Helsinki Declaration, and patient information was de-identified before analysis according to the Helsinki Declaration’s criteria.

### 2.2 Variable extraction and data pre-processing

Following ethical approval and consent from the hospital’s information technology department, experts from the information department accessed participants’ electronic medical records for the clinical study and gathered the following information. Information identifying specific individuals may be collected during the data collection procedure. However, the data collection information specialists did not participate in the subsequent data processing and statistical analysis and instead substituted serial numbers for personal information. As a result, the anonymity of the ensuing analysis may be guaranteed. As a result, the anonymity of the subsequent analysis may be guaranteed.

Comprehensive clinical data were retrospectively collected through standardized case report forms, including four key domains: (1) Clinical characteristics comprising demographic parameters (age and sex), comorbidities/history of disease (including a history of VTE prior to admission, hypertension, diabetes, stroke, severe acute pancreatitis, acute myocardial infarction, rheumatic diseases, coronary heart disease (CHD), malignant neoplasms, and sepsis), and thromboembolic risk factors (recent surgical history within 1 month and bedridden for at least 3 days); (2) Signs and Symptoms documented through physical examination findings (lower limb edema and leg circumference) and validated risk assessment instruments, specifically the Caprini Risk Score, Glasgow Coma Scale (GCS), and Acute Physiology and Chronic Health (APACHE) II; (3) Laboratory index encompassing inflammatory and nutritional biomarkers (procalcitonin and white blood cell and albumin) and coagulation function tests [thrombin time (TT), prothrombin time (PT), activated partial thromboplastin time (APTT), D-dimer, fibrinogen, and platelet]; (4) Therapeutic interventions categorized as pharmacologic management (hormone therapy, vasoactive drugs, sedatives) or procedural care (Central Venous Catheter (CVC), Invasive Blood Pressure (IBP), blood transfusion and continuous renal replacement therapy (CRRT). All measurements followed institutionally approved protocols with predefined operational definitions.

### 2.3 Definition of outcome

HA-VTE was defined as DVT or PE occurring at least 48 h after clinical admission or within 90 days of hospital discharge following an inpatient stay of at least 2 days (1). HA-VTE events occurring in critically ill patients from 48 h after mechanical ventilation to 48 h after extubation were considered outcome events in this study. The primary outcome was objectively confirmed hospital-acquired VTE by imaging studies, encompassing both symptomatic and asymptomatic DVT and PE, as clinically ordered by the treating physicians. Specifically, DVT was diagnosed by compression ultrasonography (22). PE was identified based on evidence of pulmonary artery obstruction or filling defects on pulmonary angiography, and thrombus was detected on CT pulmonary angiogram (CTPA) (23). All imaging results were reviewed and verified by board-certified radiologists.

### 2.4 Statistical analysis

Statistical analyses and graphics were performed using the SPSS statistical software (version 23.0; IBM Corp., Armonk, NY, United States) and R software (version 3.1.2; The R Foundation for Statistical Computing, Vienna, Austria) with the RMS statistical packages. The quantitative data obeying normal distribution were expressed as the mean. Standard deviation (s), and comparisons between groups were made using the *t*-test: the quantitative data not normally distributed were expressed as the median (interquartile) [M (Q1–Q3)], and comparisons between groups were made using the Mann–Whitney *U-*test. Frequencies are reported as n (%) and analyzed across groups via the Chi-square test or Fisher’s exact test as appropriate. A univariate logistic regression analysis was performed to identify predictors of HA-VTE in mechanically ventilated ICU patients. Consequently, factors with a *p*-value < 0.05 in the univariate analysis were incorporated into a multivariate logistic regression analysis (employing a backward stepwise technique) to identify independent predictors, resulting in a nomogram to estimate HA-VTE risk within this population. Regression coefficients were used as weights in the prediction model. Before developing the multivariable regression model, multicollinearity among predictors was assessed using variance inflation factors (VIFs), with variables exceeding the threshold of VIF > 5 being systematically excluded from subsequent analyses. The independent risk factors were analyzed using R4.1.2 software, and the rms program package was used to construct the column-line graph model. The receiver operating characteristic curve (ROC) assessed the model’s efficacy. Since the concordance index (C-index) is analogous to the AUC in logistic regression, we utilized the AUC to determine the discriminative performance of the nomogram. Internal consistency was verified through 1,000 bootstrap samples, and model accuracy was evaluated with a calibration curve alongside the Hosmer–Lemeshow test for goodness of fit. Decision curve analysis (DCA) was performed to ascertain the model’s clinical utility. A *p*-value of < 0.05 was considered statistically significant for all analyses.

## 3 Results

### 3.1 Baseline demographics and clinical characteristics

**FIGURE 1:**
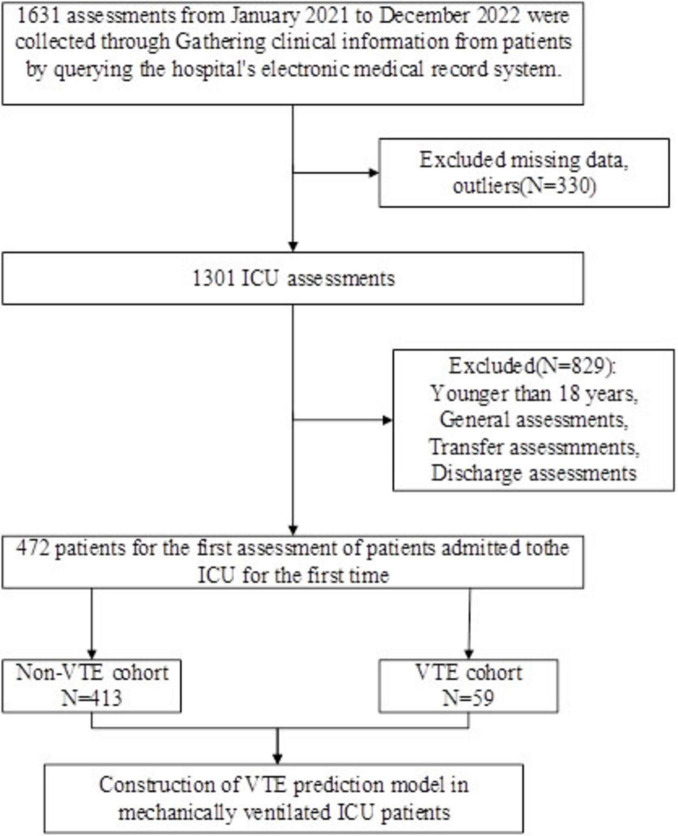
Screening flow chart of research object screening. ICU, intensive care unit; VTE, venous thromboembolism.

We collected a sample of 1,631 assessments, leaving 1,301 after excluding missing data, outliers, etc. Only data from the first assessment of a patient’s first admission to the ICU were included, after establishing inclusion and exclusion criteria, we assembled a cohort of 472 mechanically ventilated ICU patients for this investigation, comprising 290 males and 182 females, with age ranging from 18 to 97 years and a median age of 68 years. During the 2-year observation period, hospital-acquired venous thromboembolism occurred in 59 individuals (Figure 1), representing 12.5% of the total cohort.

The comparative analysis demonstrated significant differences between the HA-VTE and non-HA-VTE groups in the following parameters *(p* < 0.05): previous history of VTE, stroke diagnosis, bedridden for at least 3 days, presence of lower limb edema, increased leg circumference, elevated Caprini Risk Score, altered Glasgow Coma Scale scores, higher D-dimer and fibrinogen levels, as well as administration of vasoactive medications and sedatives. Conversely, no significant differences were observed between the two groups regarding the following variables *(p* > 0.05): Demographics such as age and gender distribution. Comorbid conditions include hypertension, diabetes, severe acute pancreatitis, acute myocardial infarction, rheumatic disease, coronary heart disease, malignant neoplasms, and sepsis. Recent surgical history within 1 month. Severity indices like APACHE II score, procalcitonin, WBC, Alb, TT, PT, APTT, and PLT. Interventions such as hormone therapy, CVC placement, IBP use, blood transfusions, and CRRT. Detailed findings are outlined in Table 1.

**TABLE 1:** Comparison of basic data between the VTE cohort and the non-VTE cohort.

Variable	VTE cohort (*n* = 59)	Non-VTE cohort (*n* = 413)	Sum (*N* = 472)	Statistic	*P*
**Clinical characteristics**					
Age [M (Q1–Q3), years]	70.00(60.00, 78.50)	67.00(53.00, 79.00)	68.00(54.00, 79.00)	–1.450	0.147
Sex (*n*%)					
Male	32(54.2%)	258(62.5%)	290(61.4%)	1.477	0.224
Female	27(45.8%)	155(37.5%)	182(38.6%)		
**Comorbidities/history of disease**					
Previous history of VTE (*n*%)	13(22.0%)	17(4.1)	30(6.4)	27.846	<0.001
YES					
NO	46(78.0%)	396(95.9)	442(93.6)		
Hypertension (n%)	33(55.9)	206(49.9)	239(50.6)	0.757	0.384
YES					
NO	26(44.1)	207(50.1)	233(49.4)		
Diabetes (n%)	19(32.2)	113(27.4)	132(28.0)	0.601	0.438
YES					
NO	40(67.8)	300(72.6)	340(72.0)		
Stroke (n%)	26(44.1)	299(72.4)	325(68.9)	21.499	<0.001
No					
Ischemic stroke	25(42.4)	74(17.9)	99(21.0)		
Hemorrhagic stroke	8(13.6)	40(9.7)	48(10.2)		
Severe acute pancreatitis (n%)				1.163	0.281
YES	2(3.4)	6(1.5)	8(1.7)		
NO	57(96.6)	407(98.5)	464(98.3)		
Acute myocardial infarction (n%)	2(3.4)	20(4.8)	22(4.7)	0.245	0.620
YES				4.650*	0.066
NO	57(96.6)	393(95.2)	450(95.3)		
Rheumatic disease (n%)					
YES	3(5.1)	5(1.2)	8(1.7)	0.140*	0.762
NO	56(94.9)	408(98.8)	464(98.3)		
CHD (n%)					
YES	4(6.8)	23(5.6)	27(5.7)	0.015	0.903
NO	55(93.2)	390(94.4)	445(94.3)		
Malignant neoplasms (n%)					
YES	5(8.5)	37(9.0)	42(8.9)	0.260*	0.489
NO	54(91.5)	376(91.0)	430(91.1)		
Sepsis (n%)					
YES	1(1.7)	4(1.0)	5(1.1)	1.046	0.306
NO	58(98.3)	409(99.0)	467(98.9)		
Recent surgical history within 1 month (n%)					
YES	14(23.7)	75(18.2)	89(18.9)	35.367	<0.001
NO	45(76.3)	338(81.8)	383(81.1)		
Bedridden for at least 3 days (n%)					
YES	41(69.5)	124(30.0)	165(35.0)		
NO	18(30.5)	289(70.0)	307(65.0)		
**Signs and symptoms**					
Lower limb edema (n%)	15(25.4)	61(14.8)	76(16.1)	4.338	0.037
YES				16.340	<0.001
NO	44(74.6)	352(85.2)	396(83.9)		
Leg circumference (n%)	29(49.2)	308(74.6)	337(71.4)		
Symmetrical				33.441	<0.001
Unsymmetrical	30(50.8)	105(25.4)	135(28.6)		
Caprini risk score (n%)					
Low-risk	3(5.1)	35(8.5)	38(8.1)	–5.111	<0.001
Medium-risk	15(25.4)	250(60.5)	265(56.1)		
High-risk	41(69.5)	128(31.0)	169(35.8)		
GCS[M (Q1–Q3), score]	7.00(5.00,8.50)	10.00(8.00,12.00)	9.00(7.00,12.00)		
APACHE II [M (Q1–Q3), score]	21.00(16.00,24.00)	19.00(13.00,25.00)	19.00(14.00,25.00)	–1.313	0.189
**Laboratory index^a^**					
Procalcitonin (n%)	34(57.6)	257(62.2)	291(61.7)	0.462	0.497
≤ 0.046 ng/mL				4.708	0.095
>0.046 ng/mL	25(42.4)	156(37.8)	181(38.3)		
WBC (n%)	1(1.7)	8 (1.9)	9(1.9)		
<4 × 10^9^/L				1.200	0.231
4∼10 × 10^9^/L	19(32.2)	194(47.0)	213(45.1)		
>10 × 10^9^/L	39(66.1)	211(51.1)	250(53.0)		
Alb (mean ± SD, g/L)	31.78 ± 6.77	30.58 ± 7.24	30.73 ± 7.18		
Thrombin time (n%)	8 (13.6)	92(22.3)	100(21.2)	2.451	0.294
<14 s				–0.7	0.487
14∼19 s	41(69.5)	252(61.0)	293(62.1)		
>19 s	10 (16.9)	69(16.7)	79(16.7)		
PT [M (Q1–Q3), s]	13.20(11.50,15.40)	12.80(11.60,14.10)	12.80(11.6,14.48)		
APTT (n%)	5(8.5)	26(6.3)	31(6.6)	1.242	0.537
< 23 s				–7.252	<0.001
23∼35 s	37(62.7)	240(58.1)	277(58.7)		
> 35 s	17(28.8)	147(35.6)	164(34.7)		
D-Dimer (mean ± SD, μg/mL)	9.69(6.00, 14.72)	3.78(1.92, 7.00)	4.16(2.19, 8.39)		
FIB (n%)	17(28.8)	279(67.6)	296(62.7)	33.134	<0.001
≤ 4 g/L				1.020	0.600
>4 g/L	42(71.2)	134(32.4)	176(37.3)		
PLT (n%)					
< 100 × 10^9^/L	16(27.1)	138(33.4)	154(32.6)		
(100∼300) × 10^9^/L	38(64.4)	247(59.8)	285(60.4)		
>300 × 10^9^/L	5(8.5)	28(6.8)	33(7.0)		
**Therapeutic intervention**					
Hormone therapy (n%)	21(35.6)	161(39.0)	182(38.6)	0.250	0.617
Yes					
No	38(64.4)	252(61.0)	290(61.4)	13.684	<0.001
Vasoactive drugs (n%)					
Yes	38(64.4)	161(39.0)	199(42.2)		
No	21(35.6)	252(61.0)	273(57.8)		
Sedatives (n%)	40(67.8)	173(41.9)	213(45.1)	13.994	<0.001
Yes				0.693	0.405
No	19(32.2)	240(58.1)	259(54.9)		
CVC (n%)	41(69.5)	308(74.6)	349(73.9)		
Yes				1.759*	0.180
No	18(30.5)	105(25.4)	123(26.1)		
IBP (n%)	3(5.1)	9(2.2)	12(2.5)		
Yes				0.044	0.834
No	56(94.9)	404(97.8)	460(97.5)		
Blood transfusion (n%)	27(45.8)	183(44.3)	210(44.5)		
Yes				0.002	0.966
No	32(54.2)	230(55.7)	262(55.5)		
CRRT (n%)	13(22.0)	90(21.8)	103(21.8)		
Yes					
No	46(78.0)	323(78.2)	369(78.2)		

*Statistically significant difference based on Fisher’s exact test (*p* < 0.05).

*^a^*Continuous variables were categorized into binary or multi-class variables using predefined clinically significant thresholds, which were determined based on established clinical guidelines or evidence-based cut-off values (as well as the index referenced from the laboratory of the First Hospital of the University of USTC). VTE, venous thromboembolism; CHD, coronary heart disease; GCS, Glasgow Coma Scale; APACHE II, Acute Physiology and Chronic Health II; WBC, White blood cell; Alb, Albumin; PT, Prothrombin time; APTT, Activated partial thromboplastin time; FIB, Fibrinogen; PLT, Platelet; CVC, Central venous catheter; IBP, Invasive blood pressure; CRRT, continuous renal replacement therapy; Mean ± standard deviation (SD).

### 3.2 Risk prediction model development

Based on univariate logistic regression analysis where VTE risk served as the dependent variable and various risk factors as independent variables, those (previous history of VTE, ischemic stroke, bedridden for at least 3 days, lower limb edema, leg circumference, Caprini Risk Score, GCS, D-dimer, FIB, vasoactive drugs, sedatives) with *p* < 0.05 underwent subsequent multivariate logistic regression analysis (detailed in Table 2).

**TABLE 2:** Univariate logistic regression analysis of risk factors for VTE in mechanically ventilated ICU patients.

Variable	ORa [95%CI*^a^*]	*P*-value
**Previous history of VTE (n%)**		
YES	6.583[3.006, 14.419]	< 0.001
NO		
**Stroke (n%)**		
No	3.885[2.121, 7.115]	< 0.001
Ischemic stroke		
Hemorrhagic stroke	2.300[0.975, 5.426]	0.057
**Bedridden for at least 3 days (n%)**		
YES	5.309[2.935, 9.603]	< 0.001
NO		
**Lower limb edema (n%)**		
YES	1.967[1.031, 3.753]	0.040
NO		
**Leg circumference (n%)**		
Symmetrical	3.100[1.819, 5.283]	< 0.001
Unsymmetrical		
Caprini risk score (n%)		
**Low-risk**		
Medium-risk	0.700[0.193, 2.541]	0.588
High-risk	3.737[1.092, 12.791]	0.036
GCS [M (Q1–Q3), score]	0.801[0.730, 0.879]	< 0.001
D-Dimer (mean ± SD, μg/mL)	1.155[1.107, 1.206]	< 0.001
**FIB (n%)**		
≤ 4 g/L	5.088[2.793, 9.268]	< 0.001
>4 g/L		
**Vasoactive drugs (n%)**		
Yes	2.921[1.635, 5.216]	< 0.001
No		

*^a^*OR, odds ratio; CI, confidence interval. VTE, venous thromboembolism; GCS, Glasgow Coma Scale; FIB, Fibrinogen; Mean ± standard deviation (SD).

**FIGURE 2:**
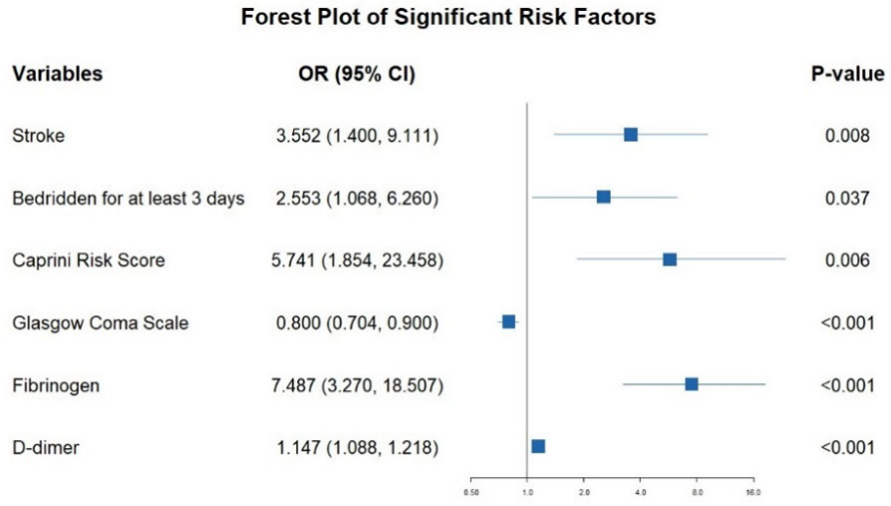
The outcomes of multivariable logistic regression analysis evaluating HA-VTE risk in mechanically ventilated ICU patients. Logistic regression model: –4.733 + 1.268 × Stroke + 0.937 × Bedridden for at least 3 days + 1.748 × Caprini risk score – 0.224 × GCS + 2.013 × FIB + 0.137 × D-dimer.

Logistic regression analysis showed that Ischemic stroke, bedridden for at least 3 days, elevated Caprini Risk Score, altered GCS scores, higher D-dimer and FIB levels are influential factors in the occurrence of VTE in mechanically ventilated ICU Patients (*p* < 0.05) (detailed in Figure 2).

### 3.3 Development of a predictive nomogram

**FIGURE 3:**
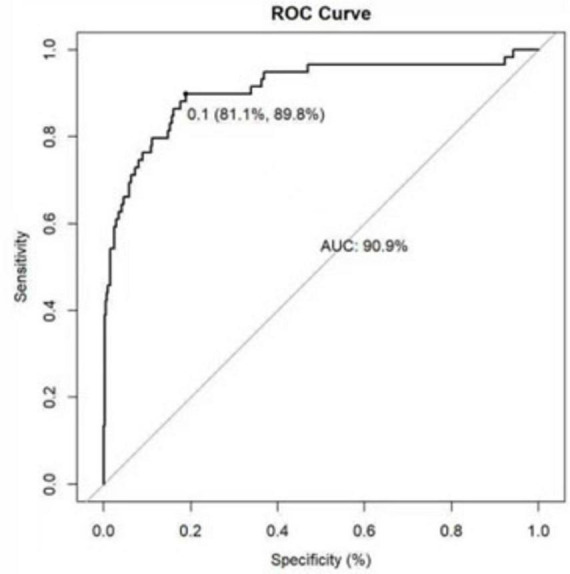
The nomogram to predict the risk of VTE in mechanically ventilated ICU patients.

A nomogram model was developed based on the results of the logistic regression analyses to evaluate the VTE risk in mechanically ventilated ICU patients. This model quantifies the influence of each identified risk factor by assigning scores. Different GCS in the figure can correspond to varying scores on Points, e.g., when the GCS score is 7, the score corresponding to Points is about 30 (if the GCS score is > 15, Points is recorded as 0); when the D-dimer level of 15 μg/mL yields 30 Points (if the D-dimer is < 0.5 μg/mL, Points is 0); when it is ischemic stroke, Points corresponds to 19 points; when the Caprini risk score is high risk, Points corresponds to approximately 39 points; and braked bedridden and plasma fibrinogen are scored as previously defined. The Points values for all six variables are summed to generate a Total Points score. This score is matched to the nearest value in the Total Points column of the scoring table. The vertically adjacent Predicted Value represents the estimated venous thromboembolism (VTE) probability, expressed as a percentage. Example: A Total Points score 100 aligns with a Predicted Value of 0.10, indicating a 10% VTE risk probability (depicted in Figure 3).

### 3.4 Validation of the nomogram

**FIGURE 4:**
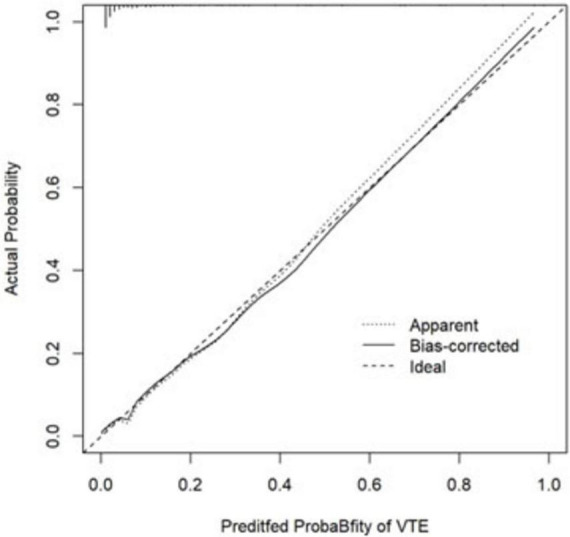
Receiver operating characteristic curve of the risk prediction model for VTE in mechanically ventilated ICU patients.

**FIGURE 5:**
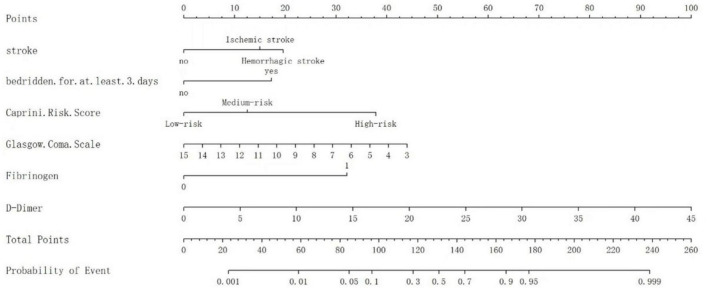
The calibration curve for predicting the probability of VTE among mechanically ventilated ICU patients. The *x*-axis depicts the predicted VTE risk, while the *y*-axis represents the actual observed incidence of VTE. The diagonal dotted line signifies perfect prediction by an ideal model, while the bias-corrected curve (solid line) is the calibration result after correcting the optimism with the 1,000 bootstrap resampling; the closer the fit to the dashed line, the more accurate the prediction.

**FIGURE 6:**
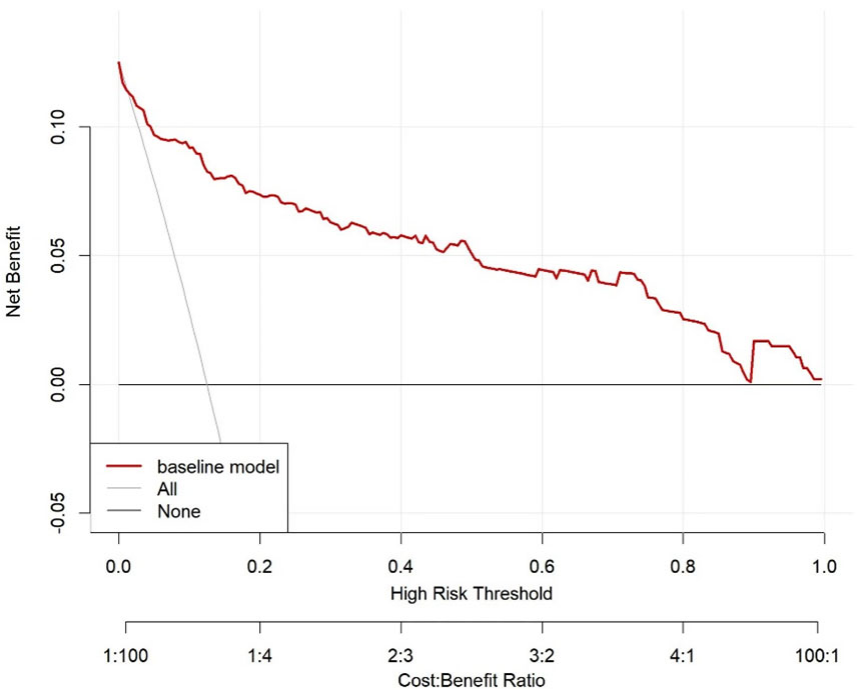
The DCA shows the clinical usefulness of the nomogram. The *Y*-axis represents the net benefit. The bold, solid red line is a nomogram predicting the risk of VTE. The solid gray line indicates that all patients experienced VTE, while the fine solid black line indicates that no patients experienced VTE. The DCA suggests that when the threshold probability falls between 0 and 99%, using this model for decision-making is more beneficial to patient outcomes than the “treat-all” or “treat-none” strategies. DCA, decision curve analysis.

The ROC curve analysis demonstrated that the AUC for the VTE risk prediction model in mechanically ventilated ICU patients stands at 0.909, with a 95% confidence interval (CI) ranging from 0.859 to 0.958 (as shown in Figure 4). This model exhibits a specificity of 81.1% and a sensitivity of 89.8%, with a Yoden index of 0.709. The model is differentiated. The alignment between predicted and observed VTE risks in this demographic is confirmed by the calibration curve (as depicted in Figure 5). The Hosmer–Lemeshow test for goodness of fit indicates a high model accuracy with a χ^2^ value of 6.398 and a *p*-value of 0.603. The DCA assesses the model’s clinical utility by quantifying net benefit—the benefit of timely intervention minus the harm of delayed intervention. The DCA validates the clinical utility of the nomogram in predicting VTE risk among mechanically ventilated ICU patients (as depicted in Figure 6). Setting the threshold probability range of the model between 0 and 0.99 reveals a net benefit above zero, substantiating the model’s efficacy.

## 4 Discussion

Mechanically ventilated patients in the ICU are at high risk for VTE (24). Among the 472 patients with mechanical ventilation enrolled in this study, 59 cases developed HA-VTE, with an incidence rate of 12.5%, which is relatively high. Data from several studies suggest that VTE occurs in 23–36.8% of mechanically ventilated critically ill patients despite preventive measures (20, 25, 26). Venous stasis results from muscular paralysis, high positive end-expiratory pressure, and injuries or occlusions of the pulmonary microvascular network in mechanically ventilated patients (27). Besides, Mechanical ventilation and positive end-expiratory pressure increase right ventricular load, decrease left ventricular load and total output, and increase the occurrence of venous blood stasis. At the same time, mechanical ventilation also alters the conversion of pulmonary fibrin, which increases coagulation and puts VTE at an increased risk of development (28). Given the high prevalence of VTE in mechanically ventilated patients and the serious consequences of its occurrence, such as physical disability and death, and according to the clinical guidelines for VTE risk stratification (29), the use of an assessment model in clinical practice is a practical and effective way to improve the management of HA-VTE prophylaxis and the selection of appropriate treatments to prevent complications. Therefore, using quantitative metrics, individualized prediction of a patient’s risk of developing HA-VTE allows for rapid identification and proper thromboprophylaxis before developing HA-VTE. Nomograms transform complex regression equations into intuitive visual graphics to accurately predict the probability of specific outcome events for individual subjects (30), making the prediction model more readable and practical. This study employed univariate and multivariate logistic regression analyses to identify independent HA-VTE risk factors in mechanically ventilated ICU patients. These factors included D-dimer, Glasgow Coma Scale, Caprini Risk Score, Fibrinogen, Stroke, and Bedridden for at least 3 days. Using these variables, a novel, simple, and practical nomogram was developed to assess HA-VTE risk. The model demonstrated excellent discriminatory capabilities, with an AUC of 0.909 (95% CI 0.859–0.958), superior to other existing models constructed by Lin et al. (31). (AUC = 0.694∼0.826) and others, along with a sensitivity of 81.1% and specificity of 89.9%, with a Yoden index of 0.709. Internal validation through 1,000 bootstrap resamplings resulted in an AUC of 0.909. The calibration curve almost overlapped with the ideal curve, and the Hosmer-Lemeshow goodness-of-fit *P*-value was 0.603, indicating that the prediction model had a high diagnostic value, and the prediction results were close to the actual probability of HA-VTE occurring in mechanically ventilated patients in the ICU, which had a good fit.

Notably, the nomogram constructed in this study, combined with the clinical net benefit confirmed by DCA, can provide precise guidance for the VTE prevention care of mechanically ventilated ICU patients. By calculating an individual’s total points and corresponding risk probability, clinicians and nurses can implement stratified management protocols to optimize resource allocation and improve outcomes. In thromboprophylaxis management, stratified protocols based on the nomogram’s risk stratification are feasible: In thromboprophylaxis intensity, the nomogram enables a protocolized escalation strategy. For a patient stratified as high-risk (for example, those with ischemic stroke, bedridden for at least 3 days, and markedly elevated D-dimer), the care pathway should adopt an escalated, consistent prophylaxis strategy. This involves nursing-led initiatives to ensure the continuous and proper application of intermittent pneumatic compression devices, with regular checks for device integrity and skin compromise, together with strict adherence to pharmacologic prophylaxis and intensified monitoring for bleeding. For patients in the moderate-to-low-risk category, standard prophylactic regimens are upheld, thus efficiently focusing intensive nursing resources on the most vulnerable individuals. For nursing vigilance and physiological monitoring, the model’s predictors provide specific targets for enhanced surveillance. High-risk patients (e.g., those with a low Glasgow Coma Scale score, a high Caprini score, or elevated fibrinogen levels) should trigger an intensified monitoring protocol. This includes systematic limb assessments for DVT signs every 4–6 h, documentation of limb circumference, and a lower threshold for ordering confirmatory Doppler ultrasonography in the event of any clinical suspicion. For low-risk patients, routine once-per-shift assessments remain appropriate, aligning with the decision curve analysis’s “risk–benefit” principle by preventing alarm fatigue and optimizing nursing workflow. Regarding early mobilization and rehabilitation, the nomogram directly informs the aggressiveness of mobility efforts. The variable “bedridden for at least 3 days” serves as a direct call to action. For a high-risk patient, this justifies immediate and sustained collaboration between the bedside nurse and physiotherapist to initiate passive range-of-motion exercises from day one, progressing to active-in-bed exercises and, eventually, upright positioning as soon as the patient’s GCS and hemodynamic status permit. For lower-risk patients, standard mobilization protocols are applied. These structured, risk-tailored measures effectively translate the DCA-confirmed net benefit into tangible clinical practice improvements, ensuring that intensive preventive strategies are focused on the highest-risk individuals, thereby systematically reducing the institutional burden of HA-VTE.

In our analysis, D-dimer concentration as the strongest predictor of HA-VTE risk in ICU patients with mechanical ventilation (OR = 1.147, *p* < 0.001), which is consistent with the findings of Chen et al. (32). For each 1 g/dL rise in D-dimer concentration, the HA-VTE incidence increase to 1.15 folds. D-dimer is a degradation product of cross-linked fibrin, a by-product of blood coagulation and catabolism. It is a laboratory indicator of coagulation function, and its elevated level reflects the presence of hypercoagulability and secondary hyperfibrinolysis (33), and helps assess individual risk of HA-VTE recurrence. A population-based nested case-control study indicates that elevated plasma D-dimer levels are associated with increased risk of incident HA-VTE (34). In clinical practice, the absence of D-dimer has often been used to rule out VTE, with a high negative predictive value (35). D-dimer testing can help rule out VTE in patients with normal D-dimer concentrations. Still, elevated concentrations do not confirm the occurrence of VTE and may also be associated with surgery, cancer, infection, or other inflammatory states (36). Thus, D-dimer is a sensitive, but not specific, indicator of VTE occurrence and is generally not used as a predictor of thrombosis alone, but usually in combination with other indicators, allowing for VTE exclusion (37). The Caprini Risk Score, a validated tool for predicting VTE risk where higher scores denote greater risk, enhances VTE risk classification in hospitalized patients (38). However, the model has fewer specific predictors regarding critically ill patients, so there are limitations in assessing the risk of developing VTE in critically ill patients (39). Furthermore, because the risk factors included in the Caprini score were mainly clinical indicators and recognized thrombotic risk factors, some known laboratory indicators that can cause thrombosis were not included in the model, making the Caprini score limited in identifying risk factors for DVT in some patients. Recent studies have shown that the predictive efficiency of Caprini increases when combined with D-dimer (40, 41), which is also demonstrated in our study. Therefore, we believe that incorporating such indicators directly into the VTE prediction models in ICU patients with mechanical ventilation will improve the model.

We also found that A higher GCS score on admission is a protective factor for VTE formation in ICU patients undergoing mechanical ventilation (OR = 0.800, *p* < 0.001). The mechanism was evident and widely recognized. GCS is a routine clinical criterion for assessing the degree of coma in patients, with 13–14 categorized as mild coma, 9–12 classified as moderate coma, and 3–8 categorized as severe coma (42). The severe paralysis or immobility on account of disturbance of consciousness caused stasis of venous flow in the lower extremity on the paralyzed side, resulting in an increased risk of thrombus being generated in the deep vein (43). As the patient’s GCS score decreases, the bedtime is also prolonged, and clinically, to protect the safety of comatose patients or the use of specific restraint methods, which affect the patient’s blood flow to varying degrees, the higher the GCS score indicates that the patient’s degree of coma is less severe, and then the patient’s risk of the formation of VTE is lower. Consequently, in comatose patients lacking voluntary movement, routine pneumatic compression of both lower limbs should be administered to proactively avoid the onset of venous thromboembolism (VTE) (44).

Fibrinogen is a crucial sign in clinical examinations. FIB is an essential protein in the blood, which plays a key role in the coagulation mechanism, helping platelets and other clotting factors to stick together tightly and form a thrombus. In ICU patients on mechanical ventilation, prolonged bed rest and poor blood circulation throughout the body may lead to hypercoagulability of the body’s blood, and the elevated level of FIB may further increase the risk of thrombus formation (45). Recent meta-analysis findings suggest that elevated fibrinogen levels are associated with an increased incidence of VTE in hip fracture patients (46). A study of 350 mechanically ventilated ICU patients demonstrated that those with elevated fibrinogen levels had a 2.675-fold higher incidence of VTE than those with normal fibrinogen levels (18). Thus, it can be inferred that FIB plays a critical role in the occurrence of VTE. Our research further indicated that FIB levels were significantly elevated in ICU patients with mechanical ventilation experiencing VTE (OR = 7.487, *p*<0.001). This discovery corroborates findings from prior studies and emphasizes the critical role of FIB levels in this patient demographic. We identified fibrinogen ≥ 4 g/L as the optimal cut-off point for predicting VTE in ICU patients on mechanical ventilation (47). Interestingly, our cut-off value is very similar to the cut-off points (3.75 g/L) in other studies on the prediction of VTE (48). The differing study populations in these two investigations—notably, the latter involving patients undergoing spinal injury surgery—suggest that elevated fibrinogen levels predict VTE across varied disease contexts.

In this study, we demonstrated that the risk of VTE increased fourfold among mechanically ventilated ICU patients with ischemic stroke (OR = 3.552, *p* = 0.008). Long-term immobilization, age, and infection are well-known risk factors for VTE, which are common in patients with ischemic stroke. In a retrospective multicenter study, among 1,632 subjects in acute ischemic stroke, 4.17% (68 subjects) had VTE (49). In 30,002 Tromsø Study participants (surveys: 1994–1995, 2001, 2007–2008), where 1,360 developed ischemic stroke and 722 developed VTE, ischemic stroke was associated with an increased VTE risk (50). Ischemic stroke, often caused by atherosclerosis, increases the risk of VTE in patients with atherosclerosis-related thrombosis through the release of inflammatory factors, which activate platelets, potentially cause endothelial damage, and promote fibrin deposition, leading to thrombosis (51). Among 1,459,865 stroke patients from one survey of the Shu study, VTE-related readmission within 90 days occurred in 0.26% (3,407/1,330,584) of Acute Ischemic Stroke patients. VTE readmission rates peaked during the initial 4–6 weeks (52). In mechanically ventilated patients, ischemic stroke may prolong ventilation duration and immobilization, potentially exacerbating venous stasis.

The model identified a substantial correlation between VTE and in-hospital immobilization based on electronic medical records tracked at the patient’s bedside. Patients on mechanical ventilation typically have minor muscle contraction or tension since they are unconscious, which can seriously worsen venous reflux. The blood stasis gets worse as the immobility period lengthens. Studies vary in how prolonged immobility is reported to raise the risk of VTE. A survey of 6,734 invasively ventilated patients enrolled from the Medical Information Mart for Intensive Care-III (MIMIC-III) database found that patients with a duration of in-hospital immobilization ranging between 4 and 7 days had an OR for VTE of 2.98 (95% CI = 2.19–4.05) while patients with a duration of more than 7 days had an OR for VTE of 6.4 (95% CI = 4.87–8.42) compared with patients immobilizing for < 4 days (31). In a retrospective study that included 2,188 consecutive neurological ICU patients, VTE was associated with a longer duration of immobilization (OR = 1.07 per day, 95% CI = 1.05–1.09) (53). In this study, we found that patients with a duration of in-hospital immobilization of more than 3 days had an OR for VTE of 2.553 (95% CI = 1.068–6.260) compared with patients immobilized for < 3 days. The meta-analysis conducted by Zang et al. (54) demonstrated that early mobilization significantly reduced ICU-acquired weakness, improved muscle strength, shortened ICU length of stay, and decreased VTE incidence.

This investigation presents several significant advantages. To the best of our knowledge, the present study first established a well-performed visualization model for HA-VTE risk prediction in ICU patients with mechanical ventilation. Unlike Lin’s study (31), we included invasive and non-invasive mechanical ventilation patients. Second, the statistical issues surrounding the development of models merit discussion. We performed statistical analyses to isolate the risk factors related to the VTE and avoided over-fitting. This model provides more accurate diagnoses and the selection of appropriate treatment methods, demonstrating robust predictive capabilities. The primary clinical utility of our nomogram lies in early identification of high-risk patients, thereby facilitating timely, tailored VTE prophylaxis. For high-risk mechanically ventilated patients identified by our model, an aggressive, multimodal prophylaxis strategy is warranted. Crucially, evidence suggests that pharmacological prophylaxis can be effective and safe even in patient populations traditionally considered at high risk for bleeding. A prospective study by Chibbaro et al. in neurosurgical patients demonstrated that a protocol combining low-molecular-weight heparin, elastic stockings, and intermittent pneumatic compression devices significantly reduced the rate of VTE without increasing the incidence of major symptomatic bleeding (55). Therefore, for high-risk mechanically ventilated patients identified by our nomogram, the clinical team should draw on this evidence to formulate and implement an intensive, multimodal prophylaxis regimen that includes pharmacological prevention, following a comprehensive assessment of the individual’s bleeding and thrombotic risks.

Nevertheless, this investigation is constrained by certain limitations. First, it is a single-center, retrospective analysis with a relatively small cohort, which may increase the risk of type II errors. Despite developing a robust nomogram model for predicting HA-VTE in ICU patients receiving mechanical ventilation, validated internally via bootstrap resampling, the lack of external validation raises concerns about its applicability across different ICU populations with mechanical ventilation. Future research should expand the sample size and employ multicenter, prospective methodologies to enhance the reliability and generalizability of the findings. Second, because all mechanically ventilated ICU patients routinely received pharmacological prophylaxis according to our unit’s standard protocol, and given the retrospective nature of this study, the specific anticoagulant type (e.g., low-molecular-weight heparin vs. unfractionated heparin) was not recorded in a structured format in our electronic medical record system. This lack of structured data directly prevented us from including and reporting the precise proportion of anticoagulant use in the present analysis. In future studies, we will designate the specific anticoagulant type and dose as a core variable to optimize data acquisition and ensure the accuracy of this information. Third, our study was conducted during the COVID-19 pandemic (2021–2022), a period during which the strong association between SARS-CoV-2 infection and venous thromboembolism became unequivocally established. A recent systematic review by Secades et al. consolidated evidence from 15 studies, confirming that COVID-19 is an independent risk factor for DVT, with reported incidences ranging from 3 to 47.5% in hospitalized patients (56). A limitation of our present study is that we did not systematically adjust for patients’ COVID-19 status in our analysis. Future studies will explicitly include and evaluate COVID-19 status as a critical predictive variable to refine model accuracy and ensure broad applicability in VTE risk prediction for ICU patients receiving mechanical ventilation.

## 5 Conclusion

This study developed and internally validated a prediction model to assess HA-VTE risk among ICU patients with mechanical ventilation. The model includes D-dimer, Glasgow Coma Scale, Caprini Risk Score, Fibrinogen, Stroke, Bedridden for at least 3 days, all demonstrating significant predictive accuracy. These easily accessible factors in clinical practice provide valuable insights for HA-VTE risk evaluation in ICU patients with mechanical ventilation.

## Data Availability

The original contributions presented in the study are included in this article/supplementary material, further inquiries can be directed to the corresponding author.
